# Comparative Analysis of Kisspeptin Levels and KISS1 Gene Polymorphism in Patients with Polycystic Ovary Syndrome (PCOS): Correlation with the Luteinizing Hormone-Follicle-Stimulating Hormone Ratio and PCOS-Associated Variables

**DOI:** 10.7759/cureus.96858

**Published:** 2025-11-14

**Authors:** Chayanika Talukdar, Saswati S Choudhary, Pinku M Talukdar, Monalisha S Borah, Achyut C Baishya

**Affiliations:** 1 Biochemistry, Gauhati Medical College & Hospital, Guwahati, IND; 2 Obstetrics and Gynaecology, Guwahati Medical College & Hospital, Guwahati, IND; 3 Multidisciplinary Research Unit, Gauhati Medical College & Hospital, Guwahati, IND; 4 Multidisciplinary Research Unit, Gauhati Medical College and Hospital, Guwahati, IND; 5 Community Medicine, Gauhati Medical College and Hospital, Guwahati, IND

**Keywords:** follicle-stimulating hormone (fsh), kisspeptin, lh/fsh ratio, luteinizing hormone (lh), single nucleotide polymorphism (snp), waist-hip ratio

## Abstract

Background: Polycystic ovary syndrome (PCOS) is one of the most common endocrine and metabolic disorders affecting women of fertile age. Its etiology is multifactorial and presumed to have a genetic basis along with environmental factors. Kisspeptins are peptide products produced from the KISS1 gene, which control the hypothalamic-pituitary-gonadal (HPG) axis and play a crucial role in human reproduction and puberty.

Objective: The study aimed to estimate the serum levels of kisspeptin, follicle-stimulating hormone (FSH), and luteinizing hormone (LH), and to investigate polymorphism in the KISS1 gene among women with PCOS and healthy controls. Furthermore, the study evaluated the association between kisspeptin and various hormonal, anthropometric, and endocrine parameters.

Methods: Two hundred women (100 with PCOS and 100 healthy controls) were enrolled in the study. Serum levels of kisspeptin, FSH, and LH were determined by enzyme-linked immunosorbent assay (ELISA). DNA was extracted, and genotyping for KISS1 was done by the polymerase chain reaction-restriction fragment length polymorphism (PCR-RFLP) method.

Results: Kisspeptin and LH waist-hip ratio (WHR) levels were significantly higher in PCOS patients when compared to the healthy control group (p value < 0.0001). Kisspeptin level was positively correlated with serum LH level (r = 0.92) and LH/FSH ratio (r = 0.45). The frequency of the CC genotype was higher in the PCOS group (p-value < 0.05). One novel single-nucleotide polymorphism (SNP, rs3924587 C>T) was detected.

Conclusion: Kisspeptin, LH, and the LH:FSH ratio were higher in patients with PCOS compared to healthy controls. KISS1 polymorphism was found to have a significant impact on various metabolic and endocrine parameters.

## Introduction

Polycystic ovary syndrome (PCOS) is an emerging and common health problem among women in the age group 12 to 45 years and has a prevalence rate that varies between 2.2% and 26% in various ethnic groups [1]. It is a significant endocrine and metabolic disorder that is characterized by chronic anovulation (amenorrhea/irregular cycles), the occurrence of polycystic ovaries on ultrasound, and the presence of hyperandrogenism (hirsutism, acne, alopecia), and is responsible for almost 75% of anovulatory infertility disorders [2,3].

Its etiopathology is complex, but it mainly results from abnormal pulsatile secretion of gonadotropin-releasing hormone [4]. The reproductive function of women depends mainly on the proper development and regulation of the hypothalamic-pituitary-gonadal (HPG) axis. From the biochemical point of view, in PCOS, the production of luteinizing hormone (LH) is excessive, and there is a normal or low level of follicle-stimulating hormone (FSH) from the anterior pituitary gland due to the ovarian dysfunction. Kisspeptins are a group of peptides expressed by the KISS1 gene, and they function through a G-protein-coupled receptor called GPR54, which takes part in regulating the HPG axis [5]. For the initiation and maintenance of mammalian fertility, the GPR54-KISS1 pathway plays a very important role. Also, kisspeptin has an important role to play in the onset of puberty, ovulation, reproduction, and fertility. It signals gonadotropin-releasing hormone (GnRH) neurons directly by its action on the kisspeptin receptor (GPR54), which releases GnRH into the portal circulation. This, in turn, stimulates the secretion of LH and FSH from the gonadotrophs of the anterior pituitary gland. In PCOS, GnRH secretion is imbalanced. So, it can be speculated that a changed level of kisspeptin may lead to an imbalanced secretion of gonadotropin in PCOS. As kisspeptin is a neuropeptide expressed from the KISS1 gene and acts through the GPR54 receptor, variation in the KISS1 gene may alter serum kisspeptin levels and hence gonadotropin secretion.

Accumulating evidence from previous studies has reported that mutations in some HPG axis-related genes like KISS1, GPR45R, GNRHR, FSHR, and LHR lead to the development of PCOS among the female adult group [6]. Among all these genes, the KISS1 gene is one of the candidate genes, which mainly regulates the female reproductive system. The KISS1 gene is localized on chromosome 1q32 and was first reported in 1996 as a metastasis-suppressing gene in a melanoma cell line. This KISS1 gene takes a critical role in ovarian cyclical activity [7]. Therefore, it becomes essential to study various mutations and the associated polymorphisms in the KISS1 gene and their role in PCOS. It has been observed that single-nucleotide polymorphisms (SNPs) in the KISS1 gene disturb the normal function of the female reproductive system by disturbing the HPG axis, and they play an important role in PCOS etiopathogenesis [8].

Similarly, for obesity, increased levels of body mass index (BMI) and waist-hip ratio (WHR) are also considered important anthropometric markers. These markers are associated with PCOS risk by directly causing disturbances in the endocrine and metabolic systems [9,10]. Also, they are responsible for some of the comorbid conditions of PCOS as reported by some studies [11,12].

To the best of our knowledge, there is a lack of Indian studies to determine any association between KISS1 gene polymorphisms and PCOS. Looking into the complicated relationship between the KISS1 gene, kisspeptin, the GPR54 receptor pathway, and the HPG axis, we have tried to identify the polymorphisms in the KISS1 gene and examine the association between KISS1 gene polymorphisms and serum kisspeptin levels, LH, FSH, the LH-FSH ratio, and WHR in women with PCOS and controls.

## Materials and methods

Recruitment of subjects

Ethical approval was obtained from the Institutional Ethics Committee, Gauhati Medical College & Hospital (GMCH), Guwahati, Assam (MC/190/2007/Pt-II/Dec-2021/14, Dated: 10/01/2022). Written informed consent was obtained from all the participants (both cases and healthy controls). The patients with well-characterized PCOS attending the obstetrics and gynecology outpatient department were recruited. The diagnosis of PCOS was made based on the Rotterdam criteria [13] and taking into consideration the diagnostic certification made by the clinical lead.

PCOS case selection was based on the following inclusion/exclusion criteria.

Inclusion Criteria

Patients who were newly diagnosed with PCOS, based on Rotterdam Criteria [13], in the age group 15-36 years were included in the study. An ultrasound scan was done in the radiology department.

Rotterdam Criteria

The patient must have at least two features out of the following three features, like oligomenorrhea (menstrual period length more than 35 days) or amenorrhea (absence of menstrual period for more than six months) [13]. Clinical and biochemical signs of hyperandrogenism, where hyperandrogenism is defined by clinical evidence of hirsutism by modified Ferriman-Gallway Score (mFG) [14] >= 8, serum testosterone (T) > 3.5 mmol/L, Free Androgen Index (FAI) > 5 [15], and polycystic ovaries on ultrasound, which is defined by a transvaginal or transabdominal ultrasound scan of ovaries performed within the first five days from the onset of menstruation & finding in at least one ovary >12 follicles measuring 2-9 mm in diameter and/or increased ovarian volume of at least 10 ml.

Exclusion criteria

Patients who have any of the following Inherited disorders of insulin resistance like Rabson-Mendenhall syndrome, known cases of hyperprolactinemia, untreated primary hypothyroidism, Cushing’s syndrome, congenital adrenal hyperplasia, androgen-secreting ovarian/adrenal tumors, pregnancy, women in the first postpartum year, women taking corticosteroids, antiepileptic, or antipsychotic drugs, and a history of hormonal contraception in the previous six months were excluded from the study.

Healthy controls

Asymptomatic, normo-androgenic, normal menstrual cycle since adolescence (menstrual period up to seven days and menstrual cycle of 21-35 days), non-medicated, consenting community-based women of reproductive age in whom PCOS was excluded by clinical, biochemical, and ultrasound assessment were considered and recruited as controls. Women of similar ethnic and social backgrounds to the affected subjects were invited to participate in the study as controls.

Sample size

The proposed study took 100 cases and 100 healthy controls. These numbers were found to be sufficient for a 95% two-sided confidence level and a power of 80, with a hypothetical proportion of controls with exposure of 50 and the least extreme odds ratio to be detected at 0.40. Calculation of sample size has been done using OpenEpi, Version 3, an open-source calculator (Dean AG, Sullivan KM, Soe MM. OpenEpi Version 3.0: Open Source Epidemiologic Statistics for Public Health. www.OpenEpi.com).

Sample collection

A specially designed questionnaire was used to collect demographic and clinical data of the patients and the control group. Age, weight, height, waist, and hip measurements, history of hirsutism, acne, and any drug or previous diseases were recorded. BMI (weight in kg divided by height in meters squared) was calculated. Also, WHR was calculated by an experienced nursing staff. Waist circumference was measured as the narrowest circumference between the lower costal margins and the iliac crest, and the hip circumference was measured as the maximum circumference at the level of the femoral trochanters with the proper standing positions of the study subjects.

In both cases and controls, blood samples were collected between day 3 and day 6 of the menstrual cycle. Participants were requested to attend the clinic at 8 am. For the test, 5 ml of blood sample was drawn from each subject using the venepuncture method. 2 ml of the collected blood was transferred to an ethylenediamine tetraacetic acid (EDTA) vial to be used for DNA extraction. The remaining 3 ml of the blood sample was taken in a serum collection vial. Serum was separated by the centrifugation process for the estimation of kisspeptin, FSH & LH. All samples were stored in a -80°C refrigerator until further experimental analysis.

Estimation of serum levels of kisspeptin, LH, and FSH

Serum level of kisspeptin was measured by using enzyme-linked immunosorbent assay (ELISA) following the manufacturer’s guidelines (BT LAB, Zhejiang, China, Cat. No. E1144Mo). For the kisspeptin kit, the intra-assay coefficient of variation (CV) is <8%, and the inter-assay CV is <10%.

Serum FSH levels were measured by using ELISA following the manufacturer’s guidelines (Qualisa, Zephyr Biomedicals, a division of Tulip Diagnostics Pvt. Ltd., Goa, India, Cat. No. 510010048). Sensitivity is 0.353; intra-assay CV% are 6.3, 5.6, and 6.7 (for levels 1, 2, and 3); and inter-assay CV% are 6.8, 6.4, and 6.2 (for levels 1, 2, and 3).

Serum LH levels were estimated by using ELISA following the manufacturer’s guidelines (Qualisa, Zephyr Biomedicals, Cat. No. 510040048). Sensitivity is 0.04, intra-assay CV% are 2.6, 3.0, and 3.4 (for levels 1, 2, and 3), and inter-assay CV% are 3.3, 3.8, and 4 (for levels 1, 2, and 3).

KISS1 genotyping analysis

Genomic DNA was extracted using a commercially available kit (Qiagen, Venlo, The Netherlands) from whole blood. The concentration of DNA was measured by Nanodrop (Thermo Fischer Scientific, Waltham, MA).

Polymerase chain reaction-restriction fragment length polymorphism (PCR-RFLP)

KISS1 polymorphism was analyzed by using the PCR-RFLP method. PCR was done, and a fragment of 318 bp was amplified using specific primer sequences: Forward: 5’- GACTCAGTGTATTCGCCCAG -3’; Reverse: 5’- GCTGGGGAGAACTCTTGAGA - 3’ (Integrated DNA Technologies, Inc., San Diego, CA, USA). The 25 μl PCR reaction mixture was prepared by adding approximately 100-150 ng of genomic DNA, 15 pmol/l of each primer, master mix (Promega, Madison, WI, USA), and deionized water (varied). The primer set was designed in-house by using Primer3 (https://primer3.ut.ee/, Cambridge, MA, USA).

The PCR cycling conditions include initial denaturation at 95°C for five minutes, followed by 35 cycles of denaturation at 95°C for 30 s, annealing at 58°C for 30 s, extension at 72°C for 45 s, and final extension at 72°C for 10 min. After amplification, the PCR products were subjected to restriction digestion using the Tsp45I enzyme (New England Biolabs, Ipswich, MA, USA). Gel electrophoresis was done, and the samples were run on 2% agarose gel and visualized in a gel documentation system. The homozygous wild-type allele has no restriction site and produces a fragment of size 318 bp (TT), whereas the heterozygous allele with a restriction site produces three fragments of size 318, 247, and 71 base pairs (CT). A homozygous allele with a restriction site produces two fragments of size 247 and 711 base pairs (CC). For internal quality control of PCR-RFLP, replication through a subset of samples was performed by randomly selecting approximately 10% of the total samples, and the results were consistent.

All the laboratory work was conducted at the Multidisciplinary Research Unit (MRU), GMCH, Guwahati, Assam. All the instruments, consumables, and equipment were provided for use by the MRU, GMCH.

Statistical analysis

All data were analyzed by the online tool OpenEpi. A p-value ≤0.05 was considered significant. The SNPStat tool (Barcelona, Spain; http://bioinfo.iconcologia.net/index.php?module=Snpstats) was used for the calculation of genotype associations and allele frequency for the SNP detected. For statistical analysis, five inheritance models (codominant, dominant, recessive, overdominant, and log-additive) were applied. The best inheritance model was assessed using the Akaike Information Criterion (AIC) [16] and the Bayesian Information Criterion (BIC) [17], and the model with the lowest values was considered and became the best fit.

## Results

**Figure 1:**
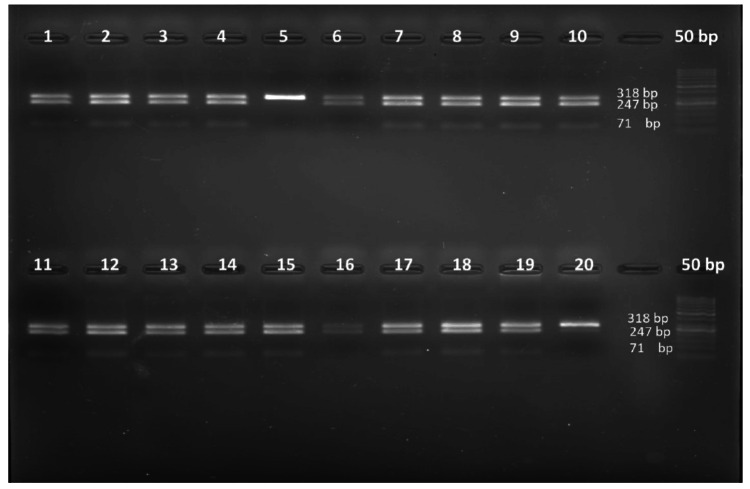
The Gel documentation image of the PCR-RFLP products PCR-RFLP: polymerase chain reaction-restriction fragment length polymorphism

The Gel documentation image of PCR-RFLP, digested by Tsp45I restriction enzyme, is shown in Figure 1. The 318 bp PCR product digested with Tsp45I is shown below. This representative image shows Lane1-4: CT, 5:TT, 6-15: CT, 16-CC, 17-19:CT, 20:CC.

For internal quality control of PCR-RFLP, replication through a subset of samples was performed by randomly selecting approximately 10% of the total samples, and the results were consistent.

For all the analyses, either the Student's t-test or the Pearson correlation coefficient was used, and the SNPStats tool was used for analyzing the genetic studies data. The demographic distribution of the study groups is presented in Table 1. There were no significant changes in age, weight, height, and BMI between the PCOS patients and the control groups. But comparatively, WHR was significantly increased in the PCOS group when compared to the control (p-value <0.05).

**Table 1:** The demographic characteristics of the PCOS patients and control PCOS: polycystic ovary syndrome

Characteristics	PCOS patients (n=100),(mean + SD )	Control (n=100), (mean + SD)	Df (Degree of freedom)	t value	p-value
Age	24.95 ± 5.25	24.12 ± 4.67	198	1.18	0.24
Weight (kg)	57.86 ± 10.29	57.07 ± 8.59	198	0.59	0.56
Height (cm)	155.22 ± 4.11	155.95 ± 4.48	198	-1.2	0.23
BMI (kg/m^2 ^)	24.13 ± 4.39	23.21 ± 2.99	175	1.73	0.09
Waist (cm)	88.53 ± 8.04	86.12 ± 8.24	198	2.09	0.04
Hip (cm)	100.99 ± 5.95	105.43 ± 8.74	100	-0.41	0.68
Waist-hip ratio (WHR)	0.88 ± 0.07	0.82 ± 0.05	179	6.97	<0.0001

Hormonal characteristics of women with PCOS and controls are shown in Table 2. Student's t-test was done, and it has been observed that women with PCOS had significantly higher serum kisspeptin and LH levels when compared to control subjects (p-value <0.0001). Also, the LH-FSH ratio was significantly higher in women with PCOS versus controls (p <0.0001). Whereas women with PCOS had serum FSH levels lower than the control subjects, and this differenceis statistically significant (p value <0.05).

**Table 2:** Endocrine characteristics of PCOS patients and control Student's t-test was done. PCOS: polycystic ovary syndrome

Characteristics	PCOS patients (n=100) (mean±SD)	Control group (n=100) (mean±SD)	Degree of freedom (Df)	t value	p-value
Kisspeptin (ng/ml)	272.43± 105.84	170.25±57.87	153	8.47	<0.0001
Luteinizing hormone (LH, IU/L)	10.56±12.50	5.40±3.25	112	3.98	0.0001
Follicle-stimulating hormone (FSH, IU/L)	4.01±3.92	8.86±4.93	188	-7.7	<0.0001
LH-FSH ratio	2.33±0.68	0.64±0.36	150	21.96	<0.0001

Pearson's correlation coefficient was calculated, and a strong positive correlation between Kisspeptin and LH (r value 0.919, p-value<0.0001) was observed (Table 3).

**Table 3:** Correlation between LH and kisspeptin in PCOS Pearson's correlation coefficient was calculated. PCOS: polycystic ovary syndrome

Parameter	R value		p-value
Luteinizing hormone (LH)	0.919	Strong positive correlation	<0.0001
Kisspeptin			

**Figure 2:**
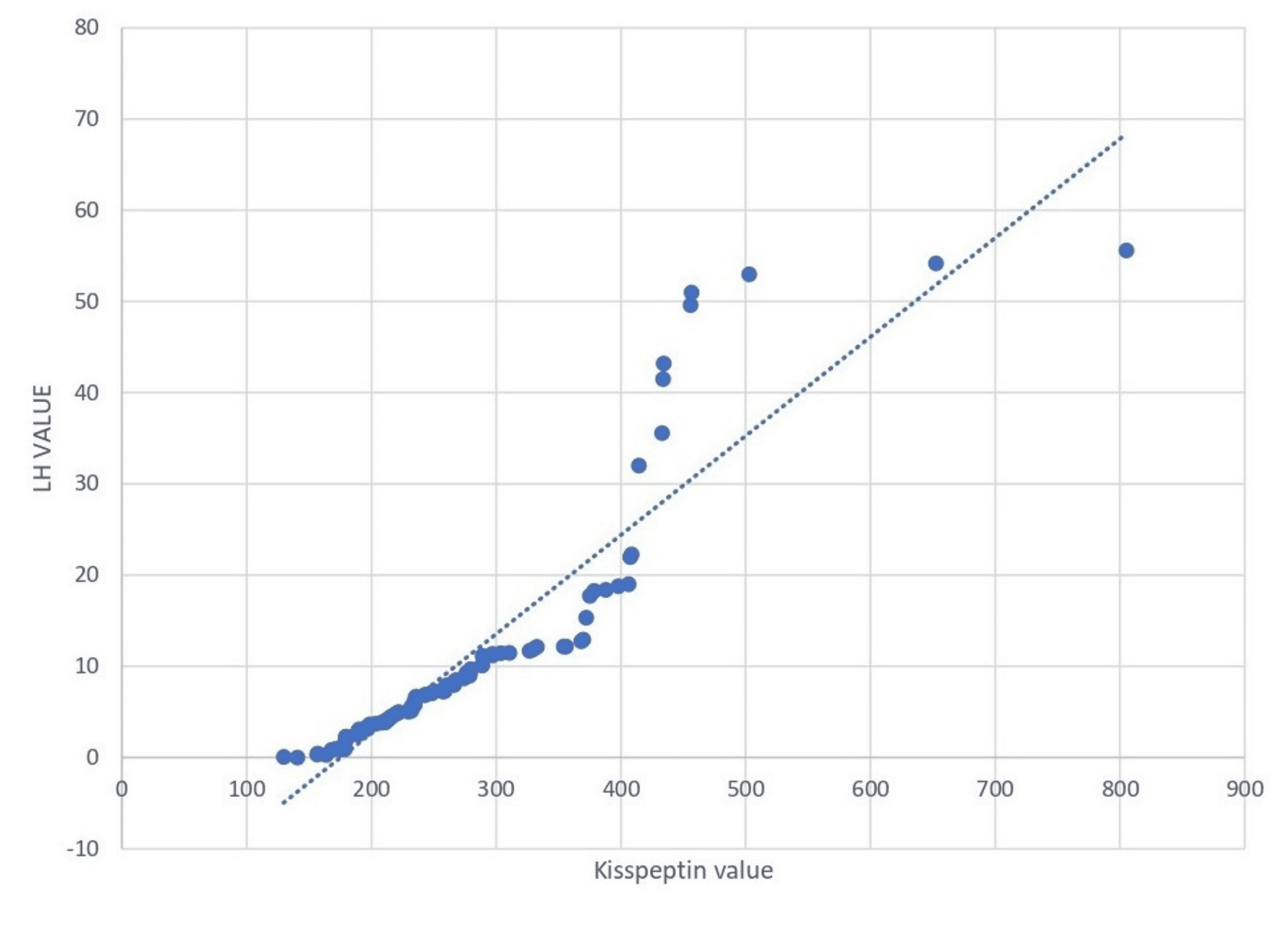
Correlation between kisspeptin and luteinizing hormone (LH)

Figure 2 shows a strong positive correlation between kisspeptin and LH (r value 0.919, p-value < 0.0001). The serum kisspeptin value is moderately positively correlated with the LH-FSH ratio (r value 0.445, p-value <0.0001), as shown in Table 4.

**Table 4:** Correlation of kisspeptin with the LH-FSH ratio in PCOS LH: luteinizing hormone; FSH: follicle-stimulating hormone; PCOS: polycystic ovary syndrome

Parameter	R value		p-value
LH-FSH ratio	0.445	Moderately positive correlation	<0.0001
Kisspeptin			

**Figure 3:**
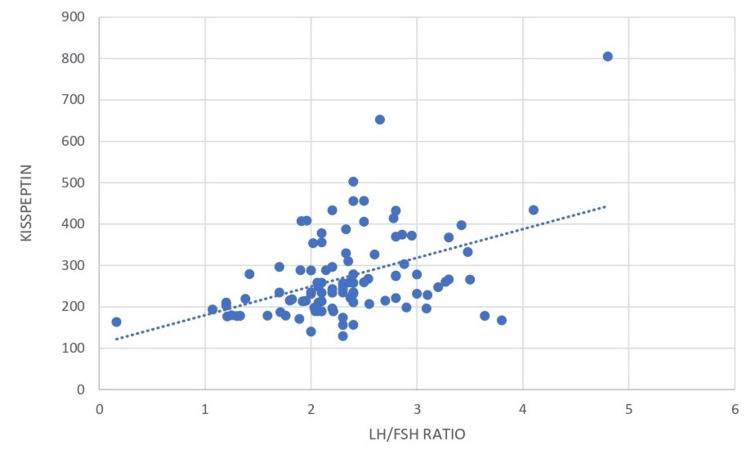
Correlation between kisspeptin and the LH-FSH ratio LH: luteinizing hormone; FSH: follicle-stimulating hormone

Kisspeptin was positively correlated with the LH-FSH ratio (r value 0.445, p-value <0.0001) as shown in Figure 3.

Gene polymorphisms were identified in the KISS1 gene analysis. PCR-RFLP of KISS1 genes revealed 1 SNP rs3924587 C>T (Table 5), located in the untranslated variant 5's prime end.

**Table 5:** Gene polymorphism of KISS1 in studied groups dbSNP ID: single-nucleotide polymorphism database ID; GRCh38: Genomic Reference Consortium Human Build 38 in 2013

Gene	Position in chromosome	Allele	Location	dbSNP ID	Amino acid change
KISS1	Chr1:204196426 (GRCh38.p14)	C>T	5-prime UTR variant	Rs3924587	N/A

Table 6 shows the genotype distribution of rs3924587 of the KISS1 gene polymorphism between PCOS cases and controls. The quantity of the heterozygous allele (CT)-there were 93 in the controls compared to 74 in the PCOS group, and the p-value (≤ 0.01) and OR (0.1910), and 95% CI indicate a significant difference. While the homozygous (TT) allele was five in the PCOS group and two in the controls, the p-value (0.4161), OR (2.0421), and 95% CI (0.3654-11.4136) indicate an insignificant difference. Again, the mutant allele (CC) number was 21 in PCOS and four in the control, with a p-value of 0.0011, and OR (6.3942) and 95% CI (6.3942) indicate a significant difference.

**Table 6:** Genotype distribution of rs3924587 of the KISS1 gene polymorphism between cases and controls Note: The number of participants in the control group is 99, as there was no amplification in one case. SNP: single-nucleotide polymorphisms

Gene	SNP	Genotype	Cases (n=100)	Control (n=99)	p-value	OR (95% CI)
KISS1	Rs3924587	CT	74 (74%)	93 (93.9%)	0.0006	0.1910 (0.0745-0.4898)
		TT	5 (5%)	2 (2.02%)	0.4161	2.0421 (0.3654 -11.4136)
		CC	21 (21%)	4 (4.04%)	0.0011	6.3942 (2.1064-19.4105)

The association of rs3924587 genotypes with PCOS under different models of inheritance is shown in Table 7. Five inheritance models (codominant, dominant, recessive, overdominant, and log-additive) were applied for the statistical analysis. The best inheritance model was assessed using the AIC and the BIC, with the model with the lowest values being the best fit (here it is overdominant).

**Table 7:** Association of SNP rs3924587 with PCOS risk under different models of inheritance Note: The number of participants in the control group is 99, as there was no amplification in one case. AIC: Akaike Information Criterion; BIC: Bayesian Information Criterion

Model	Genotype	Cases (n=100)	Control (n=99)	OR (95% CI)	P-value	AIC	BIC
Co-dominant	CC	21 (21%)	4 (4.04%)	1.00	0.0003	265.7	275.6
	CT	74 (74%)	93 (93.9%)	6.60 (2.17-20.06)			
	TT	5 (5%)	2 (2.02%)	2.10 (0.30-14.87)			
Dominant	CC	21 (21%)	4 (4.04%)	1.00	0.0002	265.7	272.3
	CT-TT	79 (79%)	95 (95.95%)	6.31 (2.08-19.16)			
Recessive	CC-CT	95 (95%)	97 (97.97%)	1.00	0.25	278.5	285.1
	TT	5 (5%)	2 (2.02%)	0.39 (0.07-2.07)			
Over dominant	CC-TT	26 (26%)	6 (6.06%)	1.00	0.0001	264.2	270.8
	CT	74 (74%)	93 (93.9%)	5.45 (2.13-13.92)			
Log-additive	-------	---------	--------	2.67(1.21-5.87)	0.01	273.3	279.9

The overdominant model showed significant differences at (0.0001) between the CC-TT and CT groups. Also, significant differences were observed in the co-dominant model for CC, CT, and TT groups at (0.0003) and in the dominant model for CC and CT-TT groups at (0.0002). In the recessive model, differences were observed for CC-CT and TT groups at (0.25), which was not significant. Finally, the log additive model also showed a significant difference (0.01).

Table 8 shows the allele frequency and allelic association of rs3924587 C>T as per the Hardy-Weinberg equilibrium law of KISS1 gene polymorphism between the patient group and the control group [18]. No significant differences were observed for the C allele at (0.1303) and the OR value (1.3586) between 0.9134 and 2.0207, and for the T allele at (0.1303) and the OR value (0.7360) between 0.4949 and 1.0948.

**Table 8:** Allele's frequency and allelic association of rs3924587 C>T by Hardy Weinberg equilibrium law of KISS1 gene polymorphism between patient group and control group Note: The number of participants in the control group is 99, as there was no amplification in one case

Alleles	Patients (n=100)		Control (n=99)		OR	95% CI	p-value
C	No	%	No	%	1.3586	0.9134-2.0207	0.1303
	116	(58.6%)	101	(51.01%)			
T	84	(41.4%)	97	(48.98%)	0.7360	0.4949-1.0948	0.1303

The influence of the rs3924587 C>T polymorphism of the KISS1 gene on the mean differences of anthropometric parameters and endocrine parameters between genotypes in patient and control groups is shown in Table 9. There are significant differences in WHR (p-value <0.0001) between cases and controls in TT genotypes. Also, there are significant differences in kisspeptin level (p-value <0.0001) and LH level (p-value <0.0001) for CT genotypes between PCOS cases and controls. while there are no significant differences for LH/FSH ratio, FSH level, weight, and BMI.

**Table 9:** Level of anthropometric parameters and endocrinal parameters in different genotypes of rs3924587 C>T in KISS1 gene with PCOS and control groups. Note: The number of participants in the control group is 99, as there was no amplification in one case. PCOS: polycystic ovary syndrome

Variable	PCOS Group (N=100) (Mean±SD)			Control group (N=99) (Mean±SD)			p-value		
	CC (N=21)	CT (N=74)	TT (N=5)	CC (N=4)	CT (N=92)	TT (N=2)	CC	CT	TT
Weight (Kg)	57.19 ±2.53	57.96 ±1.16	59.2 ±4.88	48.25 ±1.25	54.08 ±1.14	53.5 ±0.5	0.27	0.87	0.15
Height (Cm)	155 ±0.78	155.23 ±0.48	156 ±3.03	158 ±3.46	157.13 ±0.5	158 ±4	0.0001	0.72	0.51
BMI (Kg/m2)	23.96 ±1.06	24.16 ±0.5	24.52 ±2.07	19.45 ±1.22	21.84 ±0.41	21.45 ±0.85	0.59	0.07	0.59
Waist-hip ratio	0.86 ±0.02	0.88 ±0.01	0.90 ±0.02	0.82 ±0.03	0.82 ±0.01	0.83 ±0	0.22	0.99	<0.0001
Follicle-stimulating hormone (FSH; IU/L)	4.37 ±0.96	4.21 ±0.53	5.86± 3.33	10.47±0.98	8.79 ±0.52	11.85±0.45	0.79	0.85	0.20
Luteinizing hormone (LH, IU/L)	10.42 ±2.37	10.44 ±1.49	12.75±7.23	6.15 ±0.87	5.42±0.35	5.55 ±0.25	0.12	<0.0001	0.05
Kisspeptin (ng/ml)	276.41±18.04	271.45±13.16	270.3±42.39	178.04±22.76	215.08±34.72	179.35±0.45	0.45	<0.0001	0.02
LH-FSH ratio	2.38± 0.12	2.33± 0.09	2.17± 0.04	0.59± 0.07	0.66± 0.04	0.47± 0.04	0.27	0.87	0.15

## Discussion

PCOS is one of the very common metabolic disorders that affects almost 10%-25% of women in the fertile age group [19]. Its etiopathogenesis is complex, based on both genetic and environmental factors.

PCOS is characterized by a disturbance in the HPG axis. Kisspeptin is a protein produced from the KISS1 gene that has a huge influence on the HPG axis activity, and it plays an important role in the human reproductive system [20]. The KISS1 gene, primarily expressed in the hypothalamus, results in the secretion of GnRH, which ultimately regulates LH and FSH secretion, and is an important genetic factor responsible for the causation of PCOS [21].

In our study, the mean age was 24.95 ± 5.25 years in the PCOS group and 24.12 ± 4.67 years in the control group. This finding was similar to a previous study done by Emekci et al. [22].

Our study observed that the mean BMI was 24.13 ± 4.39 kg/m² in the PCOS group and 21.69 ± 3.86 kg/m² in the control group, which was not statistically significant (p>0.05). Ibrahim et al's study found results that were similar to our study [23].

In our study, the mean WHR was 0.88 ± 0.07 in patients with PCOS and 0.82 ± 0.05 in the control group, which was significant (P<0.0001). These findings are consistent with the results of some other recent studies [24, 25]. Abdominal obesity, compared to general obesity, has special importance in predicting women with PCOS [22].

In our study, the mean serum LH and LH-FSH ratios were higher in PCOS patients (p-value < 0.0001) when compared to the control group. This finding was similar to Daghestani et al. [25]. Risvanli et al. suggested that kisspeptin, by acting on GnRH, increased the LH, and the LH value was higher in women with PCOS [26].

In the present study, kisspeptin levels were higher in PCOS women as compared with the normal women (p < 0.0001). This finding was consistent with many studies [13, 23-24]. Some other studies did not obtain this variation [19-21]. Gorkem et al. [27] and Araujo et al. [4] compared kisspeptin levels in women with PCOS and with controls. They revealed that the serum kisspeptin levels were higher in women with PCOS than in controls. Liu et al. [28], in their meta-analysis, reported that serum kisspeptin levels were higher in PCOS patients than in controls, and kisspeptin might be a potential biomarker of PCOS. Another study reported a cut-off level of kisspeptin as 189 pg/mL [29]. Perez Lopez et al. [30], İbrahim et al. [23], and Umayal et al. [7] reported that women with PCOS had higher kisspeptin levels than controls in their studies, which was significant. Also, they reported that increased kisspeptin levels could be used as an early marker of PCOS to recognize it from adolescence. McCarthy et al. reported that kappa receptor agonists decrease the level of LH by inhibiting Kiss1 neurons in mice [31]. Therefore, they concluded that PCOS can be treated by suppressing LH activity through the activation of kappa receptors. But some studies, like Emekçi Özay et al. [22], Panidis et al., and Albalawi et al. [8], reported in their studies that the serum levels of kisspeptin did not differ significantly between PCOS and healthy women.

In our study, serum kisspeptin was significantly correlated with serum LH level (r=0.919, p <0.0001) and also with the LH-FSH ratio (r=0.445, p <0.0001). As per the reported literature, it was consistent with the idea that kisspeptin stimulates LH secretion. Similarly, Chen et al. reported that LH levels of both adult and adolescent PCOS women were higher than those of adolescent controls, and kisspeptin showed a positive correlation with LH. But Panidis et al. reported that no significant correlation between plasma kisspeptin and LH levels was observed in their study. Some studies showed evidence that kisspeptin may be an important factor in the activation of the HPG axis at puberty and may act as a marker to diagnose PCOS at an earlier stage. In contrast, as per Jeon et al. [32], there were no correlations between kisspeptin and any of the hormones. Yilmaz et al. [33] found that kisspeptin levels were higher in PCOS than in controls, and also a positive correlation between kisspeptin and LH levels in the PCOS group, which was similar to our study. Whereas some studies have shown that there is no significant difference in the level of kisspeptin in women with PCOS as compared to the control group [22, 34]. There may be many potential reasons for conflicting findings across various studies, like differences in assay methods, population characteristics, BMI stratifications, etc.

We also evaluated the polymorphism in the KISS-1 gene, which may act as a marker of PCOS and its risk factor. In this study, one novel SNP (rs3924587 C>T) in the KISS1 gene was identified and studied. The effect of this SNP was investigated by evaluating and comparing genotype groups, and it was observed that this led to disturbances and variations in the anthropometric and endocrine parameters (WHR, kisspeptin, LH, FSH, and LH-FSH ratio) in the PCOS group when compared to the control group.

In this study, the results showed that the SNP rs3924587 C>T was located in the 5' untranslated region (UTR) of the KISS1 gene. The 5' UTR acts as an important factor in the regulation of gene expression by regulating the process of mRNA stability, translation initiation, mRNA secondary structure, and binding of regulatory proteins or miRNA. The process of gene expression mainly depends on this initiation and translation step. Therefore, we can hypothesize that the rs3924587 C>T polymorphism of the KISS1 gene can imbalance the multi-step process of gene expression, and this can cause disturbance in the functional activity of the KISS1 gene product, like kisspeptin and its receptor GPR54.

The genotypes and allele frequencies were calculated and compared between PCOS cases and controls to elucidate and evaluate the role of the studied SNP in PCOS. The genotypes CT and CC showed a significant association with the risk of PCOS. The frequency of the CC genotype was significantly higher in PCOS patients, and the frequency of the CT genotype was significantly higher in the control subjects. When the results in different genotypes were compared, WHR was higher in the TT genotype. The level of LH and kisspeptin in the PCOS group was significantly higher in the CT genotype when compared to the controls with the same genotype. Likewise, weight, BMI, and LH/FSH ratio were higher in all genotypes in the PCOS group compared to the control group, but the results were not statistically significant. The frequency of allele C was higher in PCOS patients compared to controls, but the result was not statistically significant (p-value 0.1303, OR 1.3586).

Kisspeptin functions as an important factor in inducing hyperandrogenism in PCOS, as reported by many studies. In the present study, we have reported the role of SNP (rs3924587 C>T) in the KISS1 gene for causing imbalance in the functional activity of kisspeptin, causing hypersecretion of LH, which in turn may be responsible for increasing the risk of PCOS. No other previous studies are available in the literature for comparing the results of our study. Therefore, the association of this polymorphism with PCOS should be confirmed by further studies, as our study, with a sample size being relatively small, does not provide strong evidence for these related findings.

Limitations of the study

As our study is a very preliminary study with a small sample size, further study with a larger sample size with a multicentric approach and functional studies linking this polymorphism to gene expression will be helpful to elucidate possible mechanisms involving KISS1 gene polymorphisms and their relationship with the expression of PCOS.

## Conclusions

We can conclude that serum kisspeptin, LH levels, and the LH/FSH ratio were significantly higher in PCOS females compared to controls. There was a significant positive correlation between kisspeptin and LH and kisspeptin and the LH/FSH ratio in PCOS patients. One novel SNP (rs3924587 C>T) in the KISS1 gene was identified and investigated in this study, which showed a considerable impact on the levels of LH, kisspeptin, and WHR in PCOS women. These findings may contribute to a better understanding of PCOS with regard to its multifactorial complex etiopathogenesis.
